# Game Plan, a Web-Based Intervention to Improve Adherence and Persistence to HIV Pre-exposure Prophylaxis and Reduce Heavy Drinking in Gay, Bisexual, and Other Men Who Have Sex With Men: Usability and User Experience Testing

**DOI:** 10.2196/31843

**Published:** 2021-11-16

**Authors:** Tyler B Wray, Philip A Chan, Christopher W Kahler

**Affiliations:** 1 Department of Behavioral and Social Sciences Brown University School of Public Health Providence, RI United States; 2 Department of Medicine Warren Alpert Medical School of Brown University Providence, RI United States

**Keywords:** pre-exposure prophylaxis, HIV, HIV prevention, mHealth, mobile health, eHealth, mobile phone

## Abstract

**Background:**

Encouraging consistent use of pre-exposure prophylaxis (PrEP) is essential for reducing HIV incidence, particularly among gay, bisexual, and other men who have sex with men (GBM), and especially those who engage in heavy drinking. Although practice guidelines recommend providing adherence counseling to PrEP patients, clinics and providers may not have the resources or expertise to provide it. Internet-facilitated interventions have been shown to improve HIV prevention outcomes, including medication and care adherence. Game Plan is a website we created to help users make a tangible plan for reducing their HIV risk. We designed additional components of Game Plan to address key individual level barriers to PrEP use.

**Objective:**

The aim of this mixed methods research is to test the usability and user experience of these components with intended users: GBM who drink heavily and are on PrEP.

**Methods:**

In study 1 (usability), we completed a detailed individual interview in which participants (n=10) walked through a prototype of the website, *thinking aloud* as they did, and completed a follow-up interview and web-based survey afterward. Study 2 (user experience) involved providing participants (n=40) with a link to the prototype website to explore on their own and asking them to complete the same follow-up survey afterward. Qualitative data were analyzed using thematic analysis, and descriptive statistics were used to analyze quantitative data.

**Results:**

Users in both studies gave the website excellent ratings for usability, overall satisfaction, and quality, and most often described the site as *informative*, *helpful,* and *supportive*. Users also rated the site’s content and feel as respectful of them and their autonomy, empathetic, and they stated that it conveyed confidence in their ability to change. The study 1 interviews highlighted the importance of the website’s esthetics to the participants’ engagement with it and its credibility in prompting genuine reflection.

**Conclusions:**

GBM who reported heavy drinking and used PrEP generally found a website focused on helping them to create a plan to use PrEP consistently to be helpful. Adopting user-centered design methods and attending to the esthetics of mobile health interventions are important steps toward encouraging engagement and reducing at-risk behaviors.

## Introduction

### Background

Medications approved for use as HIV pre-exposure prophylaxis (PrEP) have considerable promise of reducing overall HIV incidence [1,2], particularly among groups who are at a higher risk of HIV such as gay, bisexual, and other men who have sex with men (GBM) [3]. Despite an increase in PrEP use in recent years, uptake of PrEP for HIV prevention is still lower than levels needed to achieve a decline in new HIV diagnoses [4]. All PrEP medications currently approved in the United States, which include Truvada, Descovy, and the generic emtricitabine-tenofovir disoproxil fumarate (FTC-TDF), are recommended for once-daily oral dosing, in part because PrEP’s efficacy in preventing HIV infection depends on consistent adherence [5]. Recent studies suggest that at least 4 doses per week provide approximately 96% efficacy [5,6]. Although many GBM using PrEP are able to achieve these levels of adherence [7-9], younger GBM may have high levels of suboptimal adherence, and they frequently discontinue using PrEP [9-12]. Interventions are needed to support GBM in successfully using PrEP while they are at risk.

Alcohol use is also an important risk factor for HIV acquisition [13], due in large part to alcohol’s tendency to interfere with effective condom use during sex in GBM [14,15]. Therefore, GBM who drink heavily are key candidates for PrEP; yet, PrEP uptake among GBM who drink heavily may be lower than in more moderate drinkers [16]. Although there is little evidence that PrEP adherence is disrupted specifically when drinking [17,18], the personal characteristics of heavy drinkers or the lifestyle disruptions they experience may be associated with suboptimal adherence, and PrEP-alcohol interactive toxicity beliefs are common [16]. As such, interventions that address alcohol’s potential role in PrEP uptake, adherence, and persistence would be valuable.

Current practice guidelines suggest that GBM follow up with their medical provider every 3 months while they are on PrEP for HIV and sexually transmitted infection (STI) testing and kidney function monitoring, among other things [19], but in practice, patients using PrEP often follow up less frequently [20,21]. Similarly, practice guidelines recommend providing adherence counseling to all patients using PrEP [19], but it is not clear how many patients using PrEP actually receive some form of counseling. It is likely that patients attending dedicated PrEP clinics are more likely to receive some form of adherence counseling, given that these clinics often have support staff available to provide it (eg, PrEP navigators) [22,23]. Although it is likely that a large proportion of PrEP patients receive their PrEP care through such specialty clinics, efforts to encourage more primary care providers to prescribe PrEP have been central to the effort to expand PrEP access [24]. The ability to provide support services such as adherence counseling is among the top barriers that primary care providers have identified to prescribing PrEP [25]. In patients using PrEP who do receive counseling, regardless of the setting, it is not clear to what extent the counseling uses techniques that are likely to improve adherence or whether it adheres to the recommendations of current practice guidelines.

Meta-analyses have shown that digital intervention programs such as websites and smartphone apps can encourage HIV prevention behaviors in users [26,27]. Alongside traditional services, these programs could be particularly useful in helping improve PrEP outcomes, given that they are relatively inexpensive and easy to disseminate [28]. Another critical advantage of digital interventions is that they can also standardize the delivery of evidence-based behavior change techniques [29]. That is, the inherently programmatic delivery of digital interventions can expose users to a consistent set of content that uses specific techniques that have been shown to help change user behavior in past studies. In this way, digital interventions can ensure that all users are shown evidence-based content and can avoid problems with fidelity and program drift that normally affect interventions delivered by individuals. Although a variety of behavioral interventions have been developed to reinforce basic PrEP counseling and increase the success rate of GBM with PrEP, few have been tested in fully powered efficacy trials to date, and nearly all are intended for face-to-face delivery [23,30-38].

Game Plan is a website we originally developed to help GBM reduce their risk of HIV and alcohol use. It was designed to align with the *spirit* of motivational interviewing (MI) [39], with content informed by the information-motivation-behavior skills model [40] and using the basic flow of many brief motivational interventions [41]. An initial pilot trial showed promising results in reducing alcohol use and the number of anal sex partners in GBM [42]. We redesigned Game Plan and expanded its content to incorporate features to help promote PrEP uptake, adherence, and persistence, as well as to help PrEP users reduce their alcohol use and risk of STIs. Thorough descriptions of its content have been reported elsewhere (Wray, TB, unpublished data, April 2021) [43]. In brief, Game Plan provides specific, digestible feedback about users’ risk of HIV given their behavior over the last year and shows them to what extent PrEP might reduce that risk with consistent adherence. It also presents information about users’ risk of other STIs, the norms of users’ sexual behaviors compared with those of other GBM in their age group, challenges several key *myths* about PrEP, and guides users through a decisional balance exercise to aid them in considering the pros and cons about their recent decisions about sex. Game Plan also guides users through various options for improving the consistency of their PrEP use and reducing their HIV and STI risk and suggests several practical steps that could help users accomplish these goals, similar to the change planning process in MI [39]. As was the case with the original Game Plan, this redesigned version provides personal feedback about users’ recent alcohol use level and its potential health consequences, including specific information about how drinking heavily could interfere with PrEP adherence or persistence, among other things.

### Objective

In this research, we employed user-centered design methods to test the usability [44] and overall user experience (UX) [45] of the redesigned Game Plan from the perspective of GBM who were currently on PrEP. Thus, this research involved conducting 2 substudies: a usability study and a UX study. In the usability study (n=10), we conducted detailed individual interviews in which participants walked through a prototype version of the redesigned site to record their thoughts and reactions to the content and note any usability issues that arose. After the interview, these participants also completed a follow-up questionnaire to rate their perceptions of the site. In the UX study (n=40), participants were provided a link to the Game Plan site through email, and after finishing all portions of it, they were asked to complete the same follow-up survey that the usability participants completed. These procedures allowed us to examine how well the redesigned website met the goals of our design process, including whether GBM on PrEP perceived that the site (1) was easy to use, (2) provided content that prompted them to reflect on their HIV and STI risk, PrEP use, and drinking, (3) aligned with the *spirit* of MI, and (4) elicited high intentions to use the site under *real world* circumstances. Although Game Plan also incorporates an SMS text messaging feature that helps support users on their progress toward the goals they set on the site, this study focused primarily on the website interaction portion.

## Methods

### Participants

A total of 50 participants were recruited from gay-oriented dating apps (eg, Grindr and Scruff) and social networking websites (eg, Facebook and Instagram) in January-March 2021 to provide feedback on a staging version of Game Plan. Of the 50 participants, 10 (20%) completed the usability portion of the study, which involved completing a baseline survey, a 60- to 90-minute usability interview over videoconference, and a follow-up survey afterward. Of the 50 participants, 40 (80%) completed the UX portion, which involved completing the same surveys as the usability participants, but they completed the follow-up survey after perusing a staging version of the Game Plan site on their own. For both the usability and UX portions, eligible participants (1) were aged ≥18 years; (2) were cisgender men; (3) could speak and read fluently in English; (4) reported anal sex with a man in the past year; (5) self-reported as currently having a valid prescription for either Truvada, Descovy, or generic FTC-TDF as HIV PrEP; (6) were prescribed to take it daily, and (7) were classified as *hazardous drinkers* according to criteria from the National Institute on Alcohol Abuse and Alcoholism, that is, they reported having consumed either ≥14 drinks per week or ≥5 drinks on a single occasion at least once in the last month [46]. Demographic characteristics for all study participants are reported in Table 1.

**Table 1:** Demographic characteristics of the usability study participants (N=50).

Characteristics		Usability test (n=10)	UX^a^ survey (n=40)
Age (range 21-68 years), mean (SD)		27.9 (3.1)	33.6 (10.9)
**Race,** **n (%)**			
	White	7 (70)	34 (85)
	Black or African American	1 (10)	2 (5)
	American Indian or Alaska native	0 (0)	0 (0)
	Asian	2 (20)	1 (3)
	Pacific Islander or Native Hawaiian	0 (0)	0 (0)
	Multiracial	0 (0)	2 (5)
	Chose not to respond	0 (0)	1.0 (3)
Ethnicity (Hispanic or Latino), n (%)		1 (10)	9 (23)
Single relationship status, n (%)		7 (70)	31 (78)
College degree, n (%)		9 (90)	35 (88)
Low income^a^, n (%)		2 (20)	8 (20)
Unemployed, n (%)		1 (10)	1 (3)
Gay or bisexual identity, n (%)		10 (100)	39 (98)
**US region of residence,** **n (%)**			
	Northeast	6 (60)	25 (63)
	South	2 (20)	3 (8)
	Midwest	1 (10)	6 (15)
	West	1 (10)	6 (15)
AUDIT^b^ total score, mean (SD)		12 (6.0)	10.8 (5.7)
DAST^c^ total score, mean (SD)		1.7 (2.5)	1.7 (1.6)
**PrEP^d^** **medication type,** **n (%)**			
	Truvada	7 (70)	28 (70)
	Descovy	3 (30)	10 (25)
	FTC-TDF^e^	0 (0)	2 (5)
Number of years on PrEP, mean (SD)		4.0 (2.0)	4.7 (1.3)
Days taken PrEP, past 30 days, mean (SD)		26.1 (6.0)	28.0 (3.6)

^a^UX: user experience.

^b^AUDIT: Alcohol Use Disorders Identification Test.

^c^DAST: Drug Abuse Screening Test.

^d^PrEP: pre-exposure prophylaxis.

^e^FTC-TDF: emtricitabine-tenofovir disoproxil fumarate.

### Procedures

Participants interested in participating were provided a link to a screening survey through email. Those who met the eligibility criteria based on the screening survey then reviewed the web-based consent information in both the bullet-point format and in the approved consent document and, if interested, indicated their consent to participate in the full studies by clicking on a radio button. Participants who consented then provided basic contact information in a subsequent survey before completing an initial baseline survey that assessed basic demographic information, relevant behavioral variables (eg, alcohol and drug use), and PrEP history. Afterward, participants in the usability portion of the project scheduled an appointment for an interview over videoconference. During this interview, participants were provided with a link to a staging version of the Game Plan site and asked to share their screen with the interviewers. They were then asked to use the site naturally and to *think aloud* as they did so. To ensure honest feedback, participants were also explicitly asked to be as critical and frank as they could be to provide us with realistic reactions. We collected video and audio recordings of the participants themselves as well as their computer screens, to allow later review of comments, clicks and navigation, and facial expressions and reactions. After reviewing all the components, staff members then conducted a brief, semistructured interview inquiring about participants’ perceptions of the app overall, including its language and tone, length and duration of its features, interactivity, ease of navigation, appropriateness, and most and least helpful components. These usability interviews lasted 60-90 minutes. Afterward, participants completed a brief web-based follow-up survey that assessed similar perceptions and reactions to the site. Participants in the UX study completed these same steps, with the exception of the usability interview portion. That is, they completed the same baseline survey but were asked to review the Game Plan site on their own, and they then completed the same follow-up survey as the usability participants. No revisions or changes to the site were made between the 2 studies. The usability study participants were compensated with US $50 for completing all portions, and the UX study participants were compensated with US $25. All procedures were approved by the Brown University Institutional Review Board.

### Measures

#### PrEP History

Items in the baseline survey collected information about participants’ PrEP use and history, including the medication they were currently taking (eg, Truvada, Descovy, or generic FTC-TDF), the date on which they were first prescribed PrEP, and self-reported daily adherence to PrEP over the last 30 days.

#### System Usability Scale

The System Usability Scale (SUS) [47] is a 10-item questionnaire that assesses a system’s ease of use, intuitiveness, and desirability. Example items include the following: *I found the various functions in this system were well integrated* and *I think that I would like to use this system frequently.*

Participants rate each item on a scale of 1 (*strongly disagree*) to 5 (*strongly agree*). The SUS has become an industry standard in the usability field, and it has excellent measurement properties (α=.90) [48]. The SUS was collected from participants in both studies as part of the follow-up survey.

#### General Usability Items and Reactions

Participants also rated several items that asked about their reactions to the site generally as well as specific aspects of it. For example, items asked participants to rate how generally *engaging, interesting* and *boring* the site was on a scale of 1 (*strongly disagree*) to 5 (*strongly agree*) and to rate the color scheme, font style, navigation, and organization of the site on a scale of 1 (*extremely disorganized* or *difficult* or *bad*) to 7 (*extremely organized* or *easy* or *good*). These items are shown in Table 2. Participants were also asked to pick up to 3 words from a list of 18 words that they believed best described Game Plan (eg, okay, helpful, terrible, all right, supportive, frustrating, unhelpful, and wonderful) and to pick and rank the components of the site that they found most and least helpful. Finally, they were also asked to rate how likely they believed that they would be to use Game Plan and its various components in their real lives (eg, email their change plan to themselves and sign up for SMS text messages to follow up on their goals) under various dissemination scenarios (eg, if a physician or a clinic provided them with a link or personally asked them to).

**Table 2:** Descriptive statistics for items rated by usability (n=10) and user experience study (n=40) participants (N=50).

Characteristics		Values, mean (SD)
**Usability or esthetics^a^**		
	Color scheme	6.0 (1.2)
	Font style	6.6 (0.8)
	Font size	6.6 (0.7)
	Navigation use	6.0 (1.4)
	Layout organization	6.3 (1.0)
**Duration, tone, and engagement^b^**		
	It was something I would want to use.	3.5 (1.1)
	It was interesting.	4.1 (0.9)
	It was engaging.	4.1 (0.8)
	It was boring.	2.1 (0.9)
	It took too long to use.	2.5 (1.1)
	I had too much on my mind to really think about the content.	2.0 (1.0)
	It was judgmental.	2.3 (1.3)
	It helped me reflect on what I do in my own life.	3.9 (0.9)
	The topics addressed were a good fit for me and other guys I know.	3.8 (0.9)
	It was *sex negative*, or makes sex seem like a bad thing.	2.1 (1.2)
**Motivational interviewing *spirit* items^b^**		
	It provided feedback objectively, rather than trying to persuade me.	3.6 (1.1)
	It told me what to do, rather than helping me understand what I want to do.	2.3 (1.0)
	It was understanding of my point of view.	3.4 (1.2)
	It showed me confidence in my ability to make changes.	3.7 (0.8)
	It showed interest in my values and goals in life.	3.5 (1.0)
	It tried to persuade me of the seriousness of the problem.	3.6 (1.0)
	It encouraged me to contribute ideas about how to change my behavior.	3.4 (1.1)
	It expressed disapproval of me.	2.2 (1.2)
	It was empathetic, meaning that it showed an understanding of my perspective.	3.6 (1.0)
	It showed respect for me.	4.1 (0.9)
	It was supportive of my ability to decide for myself.	3.8 (0.8)
	It was caring and friendly.	4.0 (0.8)
	It was honest and trustworthy.	3.9 (0.9)

^a^Items rated on a scale of 1 (*extremely bad* or *difficult* or *disorganized*) to 7 (*extremely good* or *easy* or *organized*).

^b^Items were rated on a scale of 1 (*strongly disagree*) to 5 (*strongly agree*).

#### Motivational Interviewing Spirit

A 13-item questionnaire was adapted from other common MI descriptions and rating systems [49-51] and was used to assess specific areas in which the app’s content and approach were consistent or inconsistent with MI style. Each item (all of which are presented in Table 2) was rated on a scale of 1 (*strongly disagree*) to 5 (*strongly agree*). In this study, we explored the summary statistics for individual items to determine specific areas for improvement that we might address in subsequent app versions.

#### The Mobile App Rating Scale: User Version

The Mobile App Rating Scale: User Version (uMARS) [52] is a 20-item rating scale that assesses the quality of mobile apps from the user’s perspective. The uMARS asks participants to rate their perceptions of a specific app or site overall and across 4 subscales: engagement, functionality, esthetics, and information quality. In preliminary studies, the uMARS has shown excellent internal consistency (α=.90) and good test-retest reliability [52]. The uMARS mean total score, subscale scores, and individual subjective quality ratings are reported here. However, item 18 (*How many times do you think you would use this app in the next 12 months if it was relevant to you?*) was omitted from total scores because repeated use was not necessarily the primary use case for Game Plan. As total scores are simply participants’ mean ratings on all assessed items, the impact of removing this item is likely to be minimal (α=.87).

#### Motivation to Change Target Behaviors

Both the baseline and the follow-up surveys assessed participants’ motivation to adhere to their PrEP and reduce their HIV and STI risk and alcohol use, using a single item reflecting participants’ stages of change related to that behavior [53]. This question asks participants to rate which statement is most true for them, with 5 response options, including the following: *I don’t see any need to [take my PrEP medication every day/reduce my drinking]* and *I’ve been [taking my PrEP medication every day/reducing my drinking] and will continue to do so*.

An additional question assessed to what extent participants would like to start changing each of these behaviors immediately, rating this on a scale of 1 (*not at all*) to 7 (*a bit*). Participants also reported whether they had made a plan to change each behavior (yes or no) and, if so, to rate how committed they were to following through with that plan on a scale of 1 (*not at all*) to 9 (*very much*).

### Analysis

All interview audiotapes were transcribed, reviewed, and coded by 2 staff members using a thematic approach to analysis [54]. Given that the purpose of these interviews was to directly inform design decisions and revisions, the staff members specifically searched for key themes about the app’s overall tone, interactivity, intuitiveness, and duration, as well as for specific feedback about the content on each screen. We also reviewed video clips of each session to note any significant facial expressions (eg, laughter, confusion, or frustration) and associated interaction behaviors (eg, clicks and data entry) that could further inform key themes. Once all transcripts were independently coded, staff members met to review them and develop consensus about consistent themes. To analyze quantitative data, we combined baseline and follow-up survey responses across both the usability and UX studies because the items assessed were the same and used descriptive statistics and pairwise 2-tailed *t* tests to compare specific items (eg, participants’ intentions to use the app if provided a link by a clinic vs their physician asking them to use it directly). All analyzed data were used to guide design adjustments to the site before deployment in planned efficacy studies and to improve knowledge about web-based intervention design and dissemination (eg, components that GBM perceive to be most helpful and+ use intentions in various scenarios). Quantitative data were analyzed in Stata 16 (StataCorp), and all procedures were approved by the Brown University Institutional Review Board (protocol number 1905002453).

## Results

### Usability Interviews

#### Numerous Themes

The usability participants’ specific suggestions on each page led to a number of important changes in Game Plan’s interfaces. The postwalk-through semistructured interviews also generally suggested that the information and exercises provided through Game Plan were largely unique and not something many users had been exposed to before, despite all participants having significant experience with PrEP (mean 4.5, SD 1.5 years):

Overall, I really enjoyed it. I'd say that the...design was done really well and that sort of helped me through. There's a lot of information especially around alcohol and HIV and the other specifics of PrEP, especially things I didn't know. So I learned something new.

Participant 6

These interviews yielded numerous other themes that could be important for PrEP and HIV interventions and, more generally, technology-facilitated interventions. Each of these themes is discussed below, with representative quotes from participants.

#### Theme 1: Esthetics and Design Were Effective in Promoting Engagement

Many participants spontaneously noted that they thought the site was well-designed and esthetically pleasing, and several explicitly tied these characteristics to their engagement with the site:

It was definitely pretty. I liked the design, I liked the color scheme. I don’t know that it was necessarily entertaining, but it was engaging the way the facts were presented along with it. I think that made it more attention-holding.

Participant 5

Stylistically, it looks great. It drew me in. I think it would draw people in. I would probably have gone all the way through it even if I were just scrolling through on my own.

Participant 4

Other participants suggested that although the site focused on health, it avoided an overly *clinical* look and incorporated an exciting and mysterious esthetic that would help pique the interests of users in this population:

The club/party design made it feel less medical and clinical. There’s almost some sexual suggestiveness that’s kind of intriguing about it. I think the sort of mysterious man in the background leads to a bit of intrigue and I think that’s what would make me look through it.

Participant 6

#### Theme 2: Credibility Is Critical for Sites That Focus on HIV and Other Health-Related Topics

Several participants commented on the importance of ensuring that a site that collected information about sexual behavior and provided information about HIV was credible. A participant noted that credibility was important to ensure that users would be willing to provide sensitive information about themselves, although (as the site onboarding explicitly highlighted) the app collected no personal information and was anonymous:

Honestly, talking about sexual practices might seem like private information and I might be a little reluctant to share information, so I think it does help that there’s like the Brown University branding on the first page, and that seems like a reputable institution so I feel comfortable moving forward.

Participant 10

Other participants mentioned the importance of credibility specifically when viewing feedback about the potential health consequences of their recent behavior. They noted that providing trusted institution branding on the site and including footnotes citing credible sources alongside any key information or results might boost user confidence in the accuracy of that information:

I saw that there was the Brown University affiliation at the bottom, so for me, that makes me feel like everything in here is going to be correct, or at least extremely likely to be correct. It was also nice to also see these citations down here that make me think that it’s more likely to be correct, although to be totally honest, I’ll probably never actually look at them.

Participant 9

#### Theme 3: Contrasting Users’ HIV Risk While Taking PrEP Daily Versus Without PrEP Made a Strong Impression About the Value of PrEP Use

Several participants commented on how calculating their personal risk of HIV based on their recent behavior and then directly comparing it what their risk would be if they consistently used PrEP versus not using PrEP elicited a strong reaction that reinforced the importance of consistent PrEP adherence:

Even while I take PrEP, I still have some reservations and fears that I might contract HIV, but I didn’t know it was that [emphasis theirs] effective. So that was a really reassuring number.

Participant 10

The massive disparity between the [HIV] risk if you take [PrEP] and the risk if you don’t, definitely was a bit of like...“Ok, I knew I should be doing it, but definitely if I miss it, I should be focusing more.”

Participant 7

These perspectives could support the notion that simply providing information about users’ HIV risk level may be insufficient for increasing motivation to use PrEP. Instead, both correcting user misperceptions about their personal risk based on their recent behavior *and* showing them *to what extent* PrEP could reduce that risk with consistent adherence could increase PrEP use motivation more directly.

#### Theme 4: Feedback About Users’ Personal Risk for STIs Given Recent Behavior Prompted Some PrEP Users to Consider Using Other Prevention Methods

Participants also noted that the specific feedback provided about their personal risk for other common STIs (gonorrhea and chlamydia) given their recent behavior also left an impression on them:

The Gonorrhea and Chlamydia numbers [also stood out to me]. It seems very high. Like I already knew it to be high, but knowing that I might contract Chlamydia from like 1 in 10 partners is pretty shocking.

Participant 10

Other participants also explicitly noted considering using other forms of prevention (eg, condoms) after viewing the feedback about their personal risk for other STIs:

I think the numbers that I saw about Chlamydia and Gonorrhea might make me more inclined to use condoms.

Participant 3

This theme suggests that providing similar feedback about users’ personal risk for STIs other than HIV may leave a similarly strong impression on some PrEP users and prompt them to consider ways to reduce their STI risk.

#### Theme 5: Users Found Content Addressing Alcohol Use in an HIV and STI Intervention Useful Rather Than Intrusive

Several participants also noted that they found the site’s content about alcohol use helpful, particularly because the role that drinking might play in increasing their sexual risk, potentially interfering with PrEP, was new to them:

The alcohol use section...I think was most helpful because it was tied to PrEP use and the risk of HIV. I had never made that kind of direct connection before.

Participant 10

Others noted that although they agreed that alcohol-focused content was helpful in terms of considering alcohol’s influence on their sexual decisions and PrEP use, they also believed it was helpful in reflecting on their alcohol use more generally. Specifically, a participant mentioned that the alcohol content helped them quantify their drinking pattern and reflect on its impact on their overall health and whether this pattern reflected their desired drinking level:

I think the alcohol section was the most helpful. I think it can be easy for me to, like, forget what the volumes that I drink are and how frequent they can become. I think understanding the volume of alcohol [was most helpful] and then also being reminded of the risks to like taking PrEP that can be associated with drinking. But I think I would probably still like to just in general try to reduce my alcohol use, even if there aren’t benefits related to my sexual health.

Participant 9

Overall, these themes largely support the conclusion that Game Plan’s design and content were engaging, digestible, and prompted the sort of reflection in some target users that we had hoped for in creating Game Plan.

### User Experience Survey

Participant ratings of individual items about the usability, esthetic characteristics, engagement, tone, and MI *spirit* are provided in Table 2. The overall mean total score of the SUS was 83.1 (SD 14.2), which corresponds to an *A* grade (on a scale of A-F) [55] and is in the top 10% of scores, suggesting excellent usability and overall satisfaction [48]. The uMARS total mean score of 4.0 (SD 0.5) further supports these findings, suggesting that users found the Game Plan site to be of above-average quality overall. Overall, participants gave the site 3.6 stars out of 5 (SD 0.8 stars) and stated that they would recommend the app to many people who might benefit from it (mean 3.3, SD 1.1).

Ratings of specific esthetic and design characteristics further support these overall findings, with participants rating Game Plan’s color scheme, font style, font size, navigation, and layout and organization positively or very positively. Participant mean ratings on the functionality subscale of the uMARS, which primarily reflects usability-type concepts such as performance, ease of use, and navigation, was 4.2 (SD 0.7). Their ratings on the esthetics subscale of the uMARS, which reflects the general appearance of the app and its graphics, was also 4.2 (SD 0.6). Of the 50 participants, 38 (76%) chose positive words to describe Game Plan overall, with informative (76%), helpful (54%), and supportive (46%) being the 3 most commonly chosen overall. Of the 50 participants, 7 (14%) chose negative words, including *patronizing, wary,* and *judgmental*, showing some clear room for improvement. Together, these findings suggest that participants generally had positive reactions to the site, found it to be of high quality, and thought that it functioned smoothly and was visually appealing to them.

Participant ratings also generally suggested that the site’s *tone* was generally at least somewhat consistent with our goals of conveying nonjudgmental, sex-positive content that helped them reflect on their own lives and was appropriate for them. Participant ratings also suggested that they perceived that Game Plan’s content and tone were at least somewhat consistent with the core aspects of the MI *spirit*, including that it was respectful of them and their autonomy to decide for themselves, that it was caring and empathetic, and that it conveyed confidence in their ability to change. However, mean ratings of other elements were lower but acceptable, including that the site was understanding of the users’ point of view and encouraged them to contribute ideas about how to change and suggesting some specific areas to improve upon.

When picking and ranking the sections of Game Plan that they found to be most helpful, participants most commonly included feedback about HIV risk (33/50, 66%), comparisons of social norms about sex (27/50, 54%), and feedback about PrEP’s impact on HIV risk (22/40, 44%) to be among the 3 most helpful components, whereas they found the pros and cons (decisional balance) exercise (24/50, 48%), change planning for sexual risk reduction (22/50, 44%), and the feedback about STI risk (20/50, 40%) to be least helpful. However, participant mean ratings on the information quality subscale of the uMARS were generally high as well (mean 4.0, SD 0.6), suggesting that most participants found that the site had high quality, relevant information that was credible, not overwhelming, and presented very well visually.

Participants also generally rated Game Plan as somewhat engaging and interesting, with these items rated above the midpoint and a mean score on the uMARS engagement subscale of 3.5 (SD 0.6). Most users also agreed that they would want to use Game Plan if they were given access in the *real world* (32/50, 64%), with 14% (7/50) suggesting that they would at least explore it. Furthermore, 46% (23/50) of the participants said that they would likely email their change plan from Game Plan to themselves afterward (13/50, 26%, possibly) and 28% (14/50) said that they would likely sign up for weekly SMS text messages to follow up on their progress toward their goals at the end of Game Plan (13/50, 26%, possibly). Interestingly, however, participant use intentions seemed to vary depending on how they were provided access to the site, with participants reporting that they believed they would be more likely to use Game Plan if their physician asked them to directly than if a clinic or physician’s office simply provided them with a link to access it (mean 3.9, SD 1.2 vs mean 3.4, SD 1.3; *t*_1_=3.6; *P*<.001). We also used pairwise *t* tests to explore whether the means of any ratings differed across minority participants versus non-Hispanic White participants or younger (aged <30 years) versus older participants, but no ratings were significantly different across these comparison groups.

## Discussion

### Principal Findings

In this study, we explored the usability and UX of a redesigned version of Game Plan among GBM who drink heavily and who were currently taking PrEP. Specific findings suggesting lack of clarity or usability problems with certain components led us to make many specific changes to the site design and interfaces before we began using Game Plan in 2 trials testing its efficacy in improving PrEP outcomes and HIV risk. For example, we revised the risk feedback to present it in a consistent format and direction (eg, HIV risk without PrEP and HIV risk with PrEP rather than to what extent PrEP reduced HIV risk) and changed how users entered certain data to increase ease of use (eg, the number of alcoholic drinks they consumed in the past 30 days), among other things. However, the results of these studies also produced important insights about internet-facilitated interventions more generally and their potential adoption among high-risk groups in the *real world*.

We identified 5 key themes from usability interviews that were frequently raised by participants. The first theme was that the site’s esthetic characteristics contributed to user interest in it. Participants consistently noted their appreciation of the appearance of the site, and some explicitly noted that this likely increased their interest in the site, their attention to it, or their perseverance with it. Engagement, which is a form of attention, voluntary absorption, or curiosity often accompanied by motivation to use a specific product [56], is essential to the success of digital health interventions in changing behavior [57,58]. That is, users will not be exposed to the components of these interventions that actively contribute to behavior change if they are not first engaged. Several studies have shown that most users disengage from health behavior change technologies within the first few days in the *real world* [59,60], a significant problem for products that depend on repeated, even daily, use to effect change. Marketing research has shown that, in addition to ease of use and usefulness, user enjoyment of apps and websites is associated with their loyalty to them [61]. Furthermore, Cyr et al [62] found that users who rated the appearance of apps as more pleasing also enjoyed these apps more, which in turn was associated with higher ratings of trust. As such, attending to the esthetic properties of websites and apps may both improve engagement and increase the credibility of its content.

The second theme further emphasized the importance of credibility and trustworthiness, especially for sites such as Game Plan that focus on topics such as HIV and other health issues. Participants noted that credibility of the content provided is essential if it is to influence user behavior. Research on alcohol interventions further supports the importance of credibility, particularly for techniques that provide users with feedback about the health consequences of behaviors or social approval of behaviors because the impact of these techniques on behavior change depends on users accepting and believing in the feedback provided [63]. Along with inattention, defensive reactions and doubt about the feedback could reduce the impact of this information on change [64-66]. Participants noted several aspects of the Game Plan design that seemed to increase its overall credibility, including prominently displaying the brand of a trusted institution and providing accessible references for key information wherever it appeared. These steps are consistent with past research showing that when evaluating the credibility of web-based information, users often check the sources of the information, who the author is, and the qualifications and credentials of the author when in doubt [67]. Designers of internet-facilitated health behavior change interventions should consider incorporating elements to enhance the credibility of feedback and information they provide users.

The third and fourth themes that emerged highlighted the value of providing digestible, specific feedback about user risk based on their recent behavior that prompted them to reflect on these decisions and the possibility of change. The third theme suggested that providing feedback to the user of the risk for HIV with and without PrEP side by side prompted participants to reflect on their recent adherence and commitment to taking PrEP every day. Past research has shown that many GBM are hesitant to start PrEP, in part because they may be underestimating their risk of HIV [68,69]. However, a brief intervention that used an HIV risk calculator to help correct recipient perceptions of their HIV risk did not seem to increase PrEP uptake in GBM [70]. Although there may be several reasons why this particular approach did not increase PrEP uptake, a possibility is that persuading GBM that they are underestimating their risk of HIV may highlight the need to take steps to reduce their risk but does not point to specific steps that would be successful. Our qualitative findings suggest that among PrEP users, illustrating the gap between their risk of HIV with and without PrEP use prompted several to reflect on the importance of consistent PrEP use. It is possible that this content could have a similar effect on GBM not yet using PrEP by illustrating to what extent they might reduce their risk of HIV if they started PrEP. Similarly, our findings show that some PrEP users noted that providing feedback about their risk of STIs other than HIV (eg, gonorrhea and chlamydia) also prompted them to consider taking other preventive steps (eg, using condoms and limiting sex partners) to reduce their risk. Encouraging PrEP users to reflect on their risk of other STIs was a key goal in redesigning Game Plan, given the high rates of STIs in those on PrEP [71,72]. Despite the clear public health relevance of reducing STIs in those on PrEP, few interventions have addressed this issue to date.

The fifth theme suggested that many participants found the alcohol content useful, both in the context of reflecting on their HIV and STI risk and on its own. In particular, the participant responses indicated that many had not considered how their drinking might contribute to their risk of HIV or affect their success on PrEP, despite a history of regular interaction with sexual health services such as HIV and STI testing and PrEP care. These findings offer support for the notion that many GBM could be open to discussing their alcohol use when receiving sexual health services [73]. Some also specifically noted the value of reflecting on their alcohol use in the context of their general health, suggesting that GBM may appreciate discussing their drinking in general medical contexts as well. Importantly, there was no indication that addressing alcohol use in this context resulted in defensiveness that might have reduced engagement in Game Plan content around PrEP use.

Findings from the UX survey provided very strong support for the overall usability, design, and appeal of the Game Plan website. The overall SUS score of 83.1 is among the top 10% scores, suggesting exceptional usability and satisfaction. Past studies also suggest that products with scores this high are most likely to produce loyalty among users, and that users are most likely to recommend products with SUS scores >80 to their friends [74]. Mean ratings of participant intention to recommend the site to their friends further support this conclusion, with most responding that they would recommend it to many of their friends. Participant mean overall *star* rating was somewhat less enthusiastic, at 3.6 out of 5 (SD 0.8 stars). Similarly, participant ratings on markers of engagement were positive overall, with participants rating the site as at least *somewhat* interesting and engaging. Although these ratings suggest that there is room for improvement in these metrics, we believe that they are quite strong for a website that was explicitly designed to challenge user beliefs and perceptions and specifically intended to do so among users who were initially relatively indifferent toward change. Perhaps most importantly, however, 4 out of every 5 users reported that they believed they would be likely to at least explore Game Plan if provided access to it outside of the research context based on branding and marketing materials alone, suggesting that the website may be capable of achieving high engagement among GBM PrEP users in the absence of incentives or other motivations. For digital health interventions, demonstrating high use intention among intended users should be a prerequisite for moving beyond the design stage.

Finally, GBM PrEP user ratings of Game Plan’s overall *tone* and alignment with the *spirit* of MI were also very encouraging and suggest that we largely met our design goals. Participant mean ratings of these items suggested that they agreed at least *somewhat* that Game Plan showed respect for them and was collaborative, empathetic, and nonjudgmental. Ensuring that Game Plan design and content aligned with these principles was essential, given that meta-analyses have identified expressing empathy and other aspects of the MI spirit (eg, collaboration and respect for autonomy) as some of the strongest potential mechanisms of MI counseling effects on health behavior change [75].

### Limitations

Although this study provides strong preliminary evidence of Game Plan’s overall usability and high engagement, several limitations should be noted. First, although the sample sizes of both the usability and UX surveys were larger than those of many other similar studies, larger sample sizes would likely yield more confidence in these findings. Second, this sample consisted largely of White, non-Hispanic, well-educated, and higher-income GBM; therefore, the results reported may differ in a more diverse sample. Third, participants were considered eligible if they self-reported currently having a valid PrEP prescription; therefore, it is possible that some participants were not on PrEP at the time of their participation. Fourth, participants were also very highly adherent to it over the past 30 days. Although Game Plan was explicitly redesigned to incorporate content to help PrEP users be successful in PrEP care, PrEP users likely inherently represent a subset of GBM who are especially motivated to reduce their risk for HIV. As such, they may be more likely to provide positive ratings in response to web-based tools that help them consider ways of reducing their HIV and STI risk, especially when compared with GBM who are less motivated to do so.

Overall, this study showed that GBM who use PrEP found the Game Plan website easy to use and engaging, and most of them reported that they would be likely to use it if a clinic or their physician provided them access to it in their real lives. The findings also suggested that the site’s overall branding and esthetic characteristics contributed to participants’ motivation to use it. The results also showed that specific Game Plan content seemed to be encouraging users to reflect on many of the issues we had intended: the value of consistent PrEP adherence for HIV risk reduction, users’ continued risk of STIs, and alcohol’s potential contribution to these issues. Now that Game Plan’s basic usability and potential for broad uptake have been established, future research is needed to test its efficacy in encouraging key outcomes in PrEP care, such as consistent PrEP adherence during periods of risk and reducing other risk factors of HIV acquisition, including STIs and heavy drinking. We are initiating trials to explore these outcomes in the coming months.

## Abbreviations

GBM: gay, bisexual, and other men who have sex with men
MI: motivational interviewing
PrEP: pre-exposure prophylaxis
STI: sexually transmitted infection
SUS: System Usability Scale
uMARS: Mobile App Rating Scale: User Version
UX: user experience
